# Rupture of the Duedenum from a Blow on the Abdomen

**Published:** 1880-06

**Authors:** Lewis S. McMurtry

**Affiliations:** Danville, Ky.


RUPTURE OF THE DUEDENUM FROM A BLOW ON THE ABDOMEN.

Two days since I was called to see a man, and while walking to the house, received the following history: The patient aged about forty-five, in good health, went out about two hours after eating his breakfast, to catch a horse which was grazing in the yard. He approached the animal from behind, and when just in the act of placing his hand upon him received a severe kick in the abdomen. The horse was heavily shod, and dealt a quick strong blow with the left hind foot. The man made his way to the house, and in fifteen minutes thereafter I saw him.

He was rolling in most intense agony, and referred the pain to the umbilical region. The extremities were cold, the pulse small, and he presented that array of symptoms which belong to shock. After placing him in bed, having his extremities rubbed with hot flannels, and administering about an ounce of whisky containing fifteen drops of the tincture of opium, reaction came on. But the pain continued, and it was a notable observation that it was, if possible, intensified by the whisky and laudanum. An examination of the abdomen disclosed no evidences of injury. The skin was neither bruised nor broken, and the abdominal muscles were in hard contraction.

A short time afterward I administered a quarter of a grain of morphia hypodermically, and continued to exhibit the drug in this manner as frequently as seemed admissible. But the patient was never free from pain. Six hours after receiving the injury he was pursuaded to take a small quantity of beef soup, but the stomach promptly rejected it.

At the end of twelve hours the patient's strength began to fail, and nineteen hours from the time of injury death released him from the intense suffering. The bladder had twice emptied itself of healthy urine, and there was no perceptible swelling of the abdomen.

Five hours after death I made a post-mortem examination, with the following result: Upon opening the abdomen a considerable quantity of bloody serum presented itself. The peritoneum was markedly injected, and lymph was already deposited upon the small intestine. Within the mesentery and within the peritoneal cavity, and surrounding the duodenum, was found a dark semi-fluid mass, which proved to be the partially digested contents of the duodenum. A careful examination of the intestine in the midst of this mass disclosed a rent into which the finger could be easily introduced. The opening was about two inches below the pylorus. Particles of beefsteak were found in the effused mass, and the whole was colored with bile and had a fresh, acid odor. Drs. Cowan and Johnstone, of this place, were present at the autopsy.

The following conclusions seem to be justified: The blow was received about two hours after the ingestion of a hearty meal. Digestion had reached that period where the point of greatest tension was in the duodenum. The blow was given to the abdomen as a whole, and the abdominal viscera were driven against the vertebral column. The rupture occurred at the point where the tension was the greatest. The autopsy also explains the increase of pain by the administration of whisky and laudanum, as these articles evidently passed through the pylorus into the peritoneal cavity. The case also illustrates the rapidity with which, under certain circumstances, inflammatory action spreads over the perineum.—Lewis S. McMurtry, M. D., of Danville, Ky., in the Louisville *Medical News*, April 27th, 1880.—*Med. and Surg. Reporter.*

